# Efficacy of Single-Dose Praziquantel for the Treatment of *Schistosoma mansoni* Infections among School Children in Rwanda

**DOI:** 10.3390/pathogens12091170

**Published:** 2023-09-17

**Authors:** Joseph Kabatende, Lazare Ntirenganya, Michael Mugisha, Abbie Barry, Eugene Ruberanziza, Emile Bienvenu, Ulf Bergman, Eleni Aklillu

**Affiliations:** 1 Department of Global Public Health, Karolinska Institutet, Karolinska University Hospital, Widerströmska Huset, 171 77 Stockholm, Sweden; josephkabatende@gmail.com (J.K.); abbiebarry9@gmail.com (A.B.); 2 Rwanda Food and Drugs Authority, Nyarutarama Plaza, KG 9 Avenue, Kigali P.O. Box 1948, Rwanda; ntirenganyal1@gmail.com (L.N.); ebienvenu3@gmail.com (E.B.); 3 College of Medicine and Health Sciences, University of Rwanda, KK 737 St., Kigali P.O. Box 3286, Rwanda; mmugisha@nursph.org; 4 Neglected Tropical Disease and Other Parasitic Disease Unit, Rwanda Biomedical Center, KG 17 Ave., Kigali P.O. Box 4285, Rwanda; ruberanzizaeugene@gmail.com; 5 Department of Laboratory Medicine, Karolinska Institutet, Karolinska University Hospital Huddinge, 141 86 Stockholm, Sweden; ulfkabergman@gmail.com

**Keywords:** praziquantel, efficacy, preventive chemotherapy, mass drug administration, school-age children, schistosomiasis, preventive chemotherapy, *S. mansoni* infection, neglected tropical diseases, Rwanda

## Abstract

Preventive chemotherapy with single-dose praziquantel is the WHO-recommended intervention strategy to eliminate schistosomiasis as a public health problem in endemic countries. Surveillance of drugs used in mass drug administration (MDA) programs is recommended to evaluate its effectiveness in reducing transmissions. After a decade-long implementation of a school-based MDA program in Rwanda, we conducted efficacy surveillance of single-dose praziquantel MDA against *S. mansoni* infection. Two weeks before MDA, stool examinations were performed to screen MDA-eligible school children (n = 4998) for *S. mansoni* infection using the Kato–Katz technique, and 265 (6.5%) children tested positive for the infection. All children received praziquantel and albendazole as preventive chemotherapy through the MDA campaign. Infected children were enrolled and followed for efficacy monitoring, and stool examination was repeated after three weeks post-MDA (n = 188). Before treatment, 173 (92%) had a light infection, and 15 (8%) had a moderate infection intensity. The primary and secondary outcomes were parasitological cure and egg reduction rates at three weeks post-treatment. The overall cure and egg reduction rates for *S. mansoni* infection were 97.9% (95% CI = 94.6–99.4) and 97.02%, respectively. Among the 173 children with light infection intensity, 170 (98.3%, 95% CI = 95.0–99.6) were cured, and among the 15 children who had moderate infection intensity, 14 (93.3%) were cured. No significant association between cure rate and pre-treatment infection intensity was observed. We conclude that single-dose praziquantel is efficacious against light-to-moderate *S. mansoni* infection. Preventive chemotherapy with praziquantel effectively reduces schistosome reservoirs and transmission among school-age children.

## 1. Introduction

Human schistosomiasis is a chronic parasitic disease caused by infection with blood flukes (trematode worms) of the genus *Schistosoma* [1]. Schistosomiasis is a public health problem in tropical and subtropical regions of Africa, Asia, the Caribbean, and South America. Globally, more than 779 million people are at risk of acquiring the infection [1]. Sub-Saharan Africa (SSA) remains the most significantly affected continent, bearing more than 90% of the global burden of schistosomiasis [2,3]. Schistosomiasis causes significant health problems with socioeconomic impact in areas with inadequate control efforts and sanitation, and most populations are impoverished [3]. School-aged children, teenagers, women, and young adults are mainly affected by the morbidity and mortality of schistosomiasis. Schistosomiasis can cause growth retardation, fatigue, weakness, impairment of memory and poor cognition, and anemia, leading to poor academic performance in infected children [2,4]. To reduce the morbidity of schistosomiasis and soil-transmitted helminths (STH), the World Health Assembly endorsed a resolution for regular treatment of all at-risk populations, particularly school-age children, through mass drug administration (MDA) of praziquantel and albendazole combination [5].

Currently, PZQ is the only approved drug recommended by the World Health Organization (WHO) for the treatment and control of all Schistosoma species worldwide [6]. The WHO recommends large-scale annual or biannual MDA with single-dose praziquantel (PZQ) preventive chemotherapy (PC) to all people at risk of infection, especially school-age children, to halt transmission of *Schistosoma mansoni* infection [6]. Neglected tropical disease (NTD) programs in many endemic countries, including Rwanda, have been implementing school-based deworming programs mainly targeting school-age children (SAC).

In Rwanda, the national NTD control program was established in 2007. In 2008, the initial disease mapping reported the overall prevalence of *S. mansoni* infection is 2.7%, ranging from 0 to 69.5% [7]. The first MDA was delivered in 2008. Despite several rounds of PC in the last decade, different studies still report a high prevalence of intestinal schistosomiasis in both pre-SAC and SAC in Rwanda, with significant variations between districts [8,9]. WHO advises member states and NTD programs to monitor drug efficacy when treatment failure is suspected or regardless of suspected drug failure when MDA is implemented for at least four consecutive years [1,10]. Studies have reported that reduced efficacy of PZQ following repeated exposure to MDA may cause threats to the effectiveness of schistosomiasis control programs as parasite tolerance or drug resistance was suspected in some studies [11,12].

To date, PZQ is the only WHO-approved drug to treat schistosomiasis for the past four decades. Its use has been scaled up at a national level as preventive chemotherapy in many endemic countries, including Rwanda. Due to the large-scale use including in children, safety and efficacy monitoring in MDA programs is recommended by WHO [10]. Despite multiple rounds of MDA with PZQ, a high prevalence of *S. mansoni* infection and reduced cure rate (CR) among school children was reported in SSA [13,14,15,16]. Currently, no confirmed evidence of PZQ resistance has been documented, but findings of drug tolerance and low CR have been reported in field studies from SSA [17,18]. Various factors, including parasite species, parasite stage, and infection intensity, influence PZQ on CR and egg reduction rate (ERR) [19]. Previous reports have indicated the need for regular monitoring of the safety and efficacy of PZQ following MDA [12,15], which is in line with WHO’s recommendations for routine efficacy monitoring of anthelmintics used in MDA [10].

Despite the implementation of MDA programs for years, the continued transmission of schistosomiasis calls for a collective and coordinated effort to control the infection. Infected but not treated individuals can serve as a reservoir for continued transmission in the community. The goal of preventive chemotherapy is treating all populations at risk through MDA at regular intervals to reduce transmission and prevent the disease. Multiple rounds of MDA are proven to reduce the disease burden over time but may also be a risk for parasite tolerance and the emergence of drug resistance. Therefore, the WHO guideline recommends continued vigilance to monitor drug efficacy over time through efficacy surveys to detect any emergence of drug resistance [1]. Efficacy surveillance in MDA programs is essential to assess the drug’s effectiveness in reducing parasite reservoir in the community to halt transmission and for early detection of parasite resistance.

The attainment of schistosomiasis control and elimination as a public health problem by 2030 requires strategic interventions, including investigating the effectiveness of PZQ used as preventive chemotherapy in MDA campaigns [1]. Periodic large-scale administration of PZQ to all at-risk populations over several years effectively reduced the disease burden in sub-Saharan Africa over time [20]. On the other hand, as the drug pressure increases, the development of parasite resistance or tolerance becomes a concern. Indeed, recent studies reported reduced effectiveness of PZQ, particularly among those with moderate to high infection intensities [13,15,21], highlighting the need to closely monitor intervention measures, including the effectiveness of drugs used in MDA programs. Although MDA has been implemented for many years, the efficacy surveillance of PZQ in MDA campaigns has not been studied in Rwanda. After a decade-long annual MDA program implementation, we conducted the first efficacy surveillance of a single-dose PZQ administered as preventive chemotherapy through MDA program by investigating both CR and ERR among infected school-age children living in the western province of Rwanda as recommended by the WHO [10].

## 2. Methods

### 2.1. Study Design, Area, and Setting

This observational prospective PZQ efficacy surveillance study was conducted during MDA camping in April 2019. The study was conducted among school-age children attending six selected schools in three districts of the western province of Rwanda. The three districts (Rubavu, Nyamasheke, and Rusizi) are located around lake Kivu and were chosen for this study based on epidemiological data related to the endemicity of *S. mansoni* infection using a purposive sampling method. Selected schools were within proximity of five (5 km) from Lake Kivu. In each district, two schools were selected based on previous schistosomiasis prevalence data, proximity to the lakes, and the number of school-age children in the school. Each school contributed a sample proportion to the whole study sample based on student population size. This was distributed to classes, and school children were systematically sampled in each class using class lists. The study area was chosen due to its high prevalence of *S. mansoni* infection as reported by previous studies, despite many rounds of PZQ MDA [22].

### 2.2. Ethical Considerations

Before the study initiation, permission to carry out the study was obtained from the relevant authorities, including education officers, health district hospitals, health centers, schoolteachers, and parents or guardians. Ethical approval to conduct PZQ efficacy surveillance was obtained from the Rwanda National Ethics Committee (RNEC) and the National Health Research Committee of the Ministry of Health, Rwanda. Study participants whose parents/guardians gave written informed consent were enrolled in the study. Children whose parents or guardians were not willing to participate were excluded from entering the study. Study participants and their guardians/parents were informed about the study and data collection processes.

### 2.3. Study Population, Treatment, and Follow-Up

All children attending the study schools were eligible for PZQ preventive chemotherapy as per the WHO guidelines and the Rwandan NTD program. Two weeks before the implementation of the MDA campaign, 4998 school children (age 5 to15 years old) attending the six study schools were screened for intestinal *S. mansoni* infection, of whom 265 (5.3%) children who were found infected. *S. mansoni*-infected children were enrolled in this PZQ efficacy surveillance study. The body weight of children was measured in kilograms (kg) and height in centimeters (cm). For assessment of the nutritional status of the children, anthropometric measurements were converted to height for age Z score (HAZ) and body mass index (BMI) for age Z score (BAZ) using the WHO Anthro-Plus software version 1.0.4 [23]. Children with HAZ and BAZ scores less than 2 standard deviations were considered stunted and wasted, respectively.

The WHO MDA guideline recommends co-administration of PZQ and albendazole (ALB) as preventive chemotherapy to control schistosomiasis and STHs, respectively [6]. Accordingly, all children attending the study schools, including our study participants, received single-dose PZQ (40 mg/kg body weight) and albendazole (400 mg) irrespective of their infection status as scheduled by the Rwandan national NTD program. Children participating in the study were given a standardized meal before the drug was administered to minimize the nauseating effect of PZQ. The study team had no role in the MDA planning or administration of the drugs. *S. mansoni*-infected children were followed to monitor the efficacy of PZQ after three weeks of MDA by analyzing the CR and ERR of *S. mansoni* infection as described below.

### 2.4. Stool Examination

Fresh stool samples were collected two weeks before receiving MDA (for *S. mansoni* infection screening) and three weeks after receiving MDA (to monitor PZQ efficacy). Two Kato–Katz smears were prepared from the collected stool sample as per the WHO recommendation [24]. Duplicate slides were prepared from each stool sample and read independently by the two laboratory technicians. Laboratory technicians from the National Reference Laboratory, Hospitals, and Health Centers analyzed samples, and senior laboratory technicians performed quality control and analyzed 10% of all stool samples examined each day. Designated study supervisors per school supervised the study enrollment, data collection, laboratory data analysis and processing, and data entry into an electronic database using tablets.

*S. mansoni* infection intensity was expressed in eggs count per gram of stool (epg) multiplied by a constant factor of 24, and then classified according to the WHO guidelines as (i) light infection (1–99 epg), (ii) moderate infection (100–399 epg), and (iii) heavy infection (epg ≥ 400) [25,26]. Parasitological examination of CR and ERR was conducted three weeks after PZQ administration as recommended by the WHO [10].

The primary study outcomes for PZQ efficacy were CR and ERR after 3 weeks of MDA. CR was defined as the proportion of egg-positive children before treatment who became egg-negative at three weeks post-MDA. The ERR was calculated as 100 times [1 − (Arithmetic mean of epg after treatment/Arithmetic mean of epg before treatment)], following the WHO guideline [10].

### 2.5. Data Processing and Statistical Analysis

All data collected in the electronic database were imported into STATA 13 (StataCorp LLC, College Station, TX, USA) for cleaning, processing, and analysis. Descriptive statistics were used to analyze the sociodemographic and baseline characteristics and presented in tables and figures. Pearson’s chi-square test or Fisher’s exact test were used to compare proportions between groups. A participant was considered negative when no *S. mansoni* egg was detected on both slides examined. Factors associated with egg count/gram of stool were analyzed using a negative binomial regression model. Predictors of CR were analyzed using univariate regression analysis. A *p*-value < 0.05 was considered statistically significant.

## 3. Results

### 3.1. Sociodemographic and Baseline Characteristics of Study Participants

**Figure 1:**
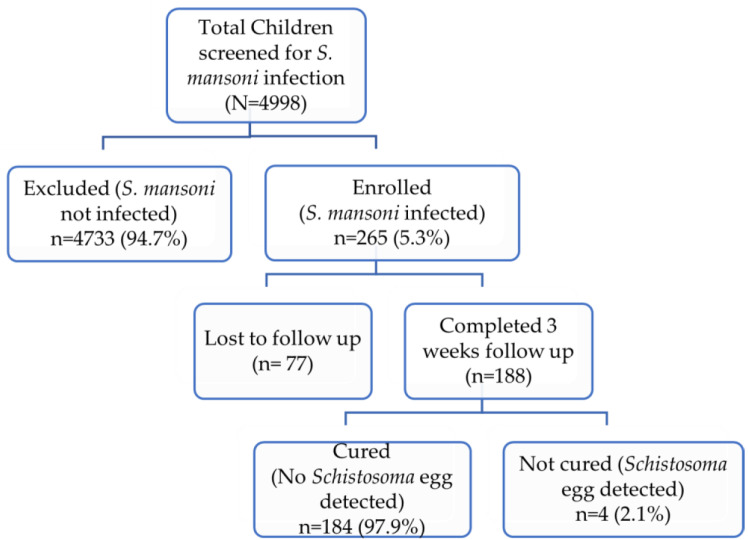
Study flow chart of participant enrollment, follow-up, and treatment outcome.

Out of the 4998 school children screened for *S. mansoni* infection, 265 tested positive and were enrolled in this PZQ efficacy surveillance study. Study participants received PZQ as part of MDA and followed up to 3 weeks to monitor treatment outcome. Only 188 (70.9%) children completed the study and 77 (29.1%) were lost to follow-up. The study flow chart, including study enrollment, follow-up, and treatment outcome after three weeks of MDA is presented in Figure 1.

The baseline sociodemographic characteristics of study participants is presented in Table 1. Most of the study participants were female (52.7%)). Of the total enrolled school-age children, 144 (76.6%) were aged from 10–15 years old, while 44 (23.4%) were aged from 5–9 years old. Most study participants had a light infection intensity (173 (92%)), while 15 (8%) had moderate infection intensity. Most of the study participants (89.4%) were coinfected with at least one type of STH parasites, and only 20 (10.6%) were not coinfected with any of the STH species screened (Hookworm, *Ascaris lumbricoides,* and or *Trichuris trichiura*) infections as described in Table 1.

### 3.2. Cure Rate and Egg Reduction Rates

WHO recommends parasitological CR and ERR to measure the PZQ drug efficacy in MDA program for schistosomiasis control and elimination [10]. The study findings revealed that CR and ERR for intestinal schistosomiasis were 97.9% and 97.0%, respectively. The ERR of 97.0% was above the WHO-recommended efficacy threshold (ERR: ≥90), as described in Table 2. The current efficacy surveillance study assessed the proportion of cured and not cured school-age children within each infection intensity after mass drug administration. The findings indicate that, among 173 cases of light infection intensity for intestinal schistosomiasis, 170 (98.3%) children were cured, while 3 (1.7%) were not cured post-drug administration. Similarly, among 15 cases of moderate infection intensity, 14 (93.3%) were cured after drug administration, while 1 (6.7%) was not cured.

### 3.3. Factors Associated with Cure Rates

The correlations of sociodemographic and baseline characteristics with CR of *S. mansoni* infection were assessed. Explorations were made on CR in each category of studied variables. Overall, 86 (96.6%) male school-age children were cured, and 98 (98.9%) female school-age children were cured after drug administration. Among 144 children aged from 10–15 years, 141 (97.9%) were cured after treatment, while among 44 school-age children (5–9 years old) who received treatment during MDA program, 43 (97.7%) were cured.

Among 60 stunted children, 59 (98.3%) were cured. Among 128 not stunted children with intestinal *S. mansoni* infection, 125 (97.7%) were cured after MDA. Among the 179 non-wasted children, 175 (97.8%) were cured, while all 9 (100%) wasted children were cured after treatment. Among the 173 children with light infection intensity of *S. mansoni* infection, 170 (98.3%) were cured, while 15 children with moderate infection 14 (93.3%) were cured. Among the 168 children co-infected with any STH infections (*Ascaris lumbricoides,* Hookworms, or *Trichuris trichiura*), 165 (98.2%) were cured, while among 20 children infected with *S. mansoni* infection, only 19 (95%) were cured.

Factors such as age, sex, consistency of stool, wasting, stunting, pre-treatment *S. mansoni* infection intensity, and co-infections with any STH infections were not significantly associated with CR of *S. mansoni* infection (Table 3).

Univariate logistic regression analysis of factors associated with CR at three weeks post-MDA of PQZ against *S. mansoni* infection revealed that factors such as age, sex, wasting, stunting, infection intensity, co-infections were not significant predictors of CR (Table 4). Since there were no biologically plausible predictor variables in the univariate analysis model, the multivariate model for analysis was not performed.

## 4. Discussion

Our study findings indicate a high CR (98%) and ERR (97%) of PZQ (Table 2), which is above the 90% threshold for satisfactory effectiveness set by the WHO [10]. The study findings show that PZQ used in MDA is effective in treating light and moderate infection intensities of *S. mansoni* infection in the study area. Consequently, MDA with PZQ effectively reduces the parasite reservoirs and transmission among school-age children, the most at-risk population for *S. mansoni* infection. The observed higher CR and ERR in our study is an indication that PZQ is efficacious against *S. mansoni* in our study setting and can still be recommended for use in MDA for the control and elimination of schistosomiasis in Rwanda. Our study finding aligns with a recent report showing that a decade-long MDA with PZQ and Albendazole in Rwanda successfully reduced the burden of schistosomiasis and STH over time [8,22].

However, despite the high MDA coverage nationwide and the good PZQ efficacy identified in this study, transmission of *S. mansoni* infection continues in the country [8]. Hence, a multisectoral approach is needed in Rwanda to achieve its NTD strategic plan and goal: to make Rwanda free from NTDs as a public health problem by 2024 by implementing WHO-recommended public health strategies for the prevention and control of NTDs [27]. Thus, implementing other preventive measures, including improving water, sanitation, and hygiene (WASH) is needed to complement the effect of MDA in controlling schistosomiasis in the country [28].

Overall, MDA with PZQ is safe and tolerable and associated adverse events are mild-to-moderate and transient, resolving within a week of receiving MDA [29,30]. However, reports on the effectiveness of PZQ in treating *S. mansoni* infection vary between populations and geographic areas [13,21,31]. Recent studies highlight genetic variations in drug-metabolizing enzymes can affect inter-individual variability in PZQ plasma exposure and treatment outcomes [32,33]. A study that evaluated the therapeutic efficacy of PZQ in seven countries indicated no overall sign of reduced effectiveness of the standard PZQ treatment, but substantial inter-individual variation in treatment responses underscored the need for efficacy monitoring [34]. Although we found high CR (98%) in our study, low CRs of PZQ, particularly from high-burden areas, are reported from Cote d’Ivoire (69%), Tanzania (81.2%), and Ethiopia (89.1%) [13,15,35]. Therefore, there is a need to continue monitoring and evaluating PZQ efficacy and inform the program implementation, policymakers, medicines regulators, and WHO for informed decision-making.

The current study indicates higher CR (98.3%) in participants with light infection intensity compared to participants with moderate infection intensity (93.3%). Our findings align with reports from Tanzania and Ethiopia, which indicated PZQ is more effective in curing light *S. mansoni* infection than moderate to heavy infections [13,15]. In our study, only 2% were not cured. This may be attributed to the poor performance of PZQ against juvenile/immature parasites, as reported in previous studies [13,36]. The findings also align with reports that treatment with PZQ reduces worm burden, intensity of infection, and reverse schistosomiasis-associated morbidity in treated individuals [37]. The revised Rwanda national NTD Strategic Plan (2019–2024) that has included regular assessment strategies for the effectiveness of drug use during MDA [27] is in alignment with the WHO global strategy that recommends regular efficacy and safety monitoring for drugs deployed during the MDA campaign [10].

Our study has some limitations. PZQ effectiveness is influenced by the burden and infection intensity of *S. mansoni* [13,15]. Though statistically not significant, we found a higher CR among light infection intensity than moderate infection intensity. In our study, most (92%) participants had light infection intensity, while only 8% had moderate infection intensity, and none had heavy infection intensity. Since most of our study participants had light infection intensity, the observed high CR and ERR may not reflect PZQ effectiveness in high *S. mansoni* infection intensity settings.

## 5. Conclusions

We report that MDA with single-dose PZQ is efficacious against *S. mansoni* infection and reduces parasite reservoirs and transmission rate among school-age children. The study findings show that PZQ in MDA reduces morbidities associated with light and moderate infection intensities caused by *S. mansoni* infection. The persistent transmission of schistosomiasis in Rwanda, despite the high MDA coverage and the good PZQ effectiveness against *S. mansoni* infection, indicates the need to equally scale up other preventive measures to consolidate gains in the control of schistosomiasis made through preventive chemotherapy. To achieve the Rwanda NTD revised strategic plan targets by 2024, intensified control and elimination measures, including a multisectoral approach that brings together the national NTD program, policymakers, partners, researchers, medical products regulators, and the community is of high importance to control morbidity and elimination of schistosomiasis in Rwanda.

## Figures and Tables

**Table 1:** Sociodemographic and baseline characteristics of study participants.

Variable		N	%
Sex	Male	89	47.3
	Female	99	52.7
Age categories	5–9 years	44	23.4
	10–15 years	144	76.6
District	Rubavu	56	29.8
	Nyamasheke	59	31.4
	Rusizi	73	38.8
School	Rambo	49	26.1
	Rubona	7	3.7
	Buhokoro	52	27.7
	Mukoma	7	3.7
	Bugumira	39	20.7
	Nkombo	34	18.1
Consistency of stool	Formed	3	1.6
	Soft	184	97.9
	Watery	1	0.5
Stunting status	Non stunted	128	68.1
	Stunted	60	31.9
Wasting status	Not wasted	179	95.2
	wasted	9	4.8
*S. mansoni* infection intensity	Light	173	92
	Moderate	15	8
	Heavy	0	0
Co-infection with STH	No	20	10.6
	Yes	168	89.4

STH = Soil transmitted helminths.

**Table 2:** Cured and egg reduction rates for *S. mansoni* infection.

S. *mansoni* Infection		Cure Status			Egg Reduction Rate (ERR)	
		Total N	Cured(n)	Cure Rate	ERR (%)	WHO Threshold
Overall		188	184	97.9%	97.0	≥90
Infection intensity	Light	173	170	98.3%	97.2	≥90
	Moderate	15	14	93.3%	96.7	≥90

**Table 3:** Association of sociodemographic and baseline characteristics with cure rates among study participants.

Variable		Schistosomiasis (n = 188)		
		N	Cured N (%)	*p*
Sex	Male	89	86 (96.6)	0.27
	Female	99	98 (98.9)	
Age categories	5–9 years	44	43 (97.7)	0.66
	10–15 years	144	14 1(97.9)	
Stunting status	Non stunted	128	125 (97.7)	0.62
	Stunted	60	59 (98.3)	
Wasting status	Not wasted	179	175 (97.8)	0.82
	wasted	9	9 (100.0)	
Infection intensity	Light intensity	173	170 (98.3)	0.28
	Moderate intensity	15	14 (93.3)	
Co-infections with STH	No	20	19 (95.0)	0.37
	Yes	168	165(98.2)	

STH = Soil-transmitted helminths.

**Table 4:** Predictors of cure rate at three weeks of post-praziquantel administrations.

Variables			Cured	Univariate Analysis		
			N (%)	cOR	95% CI	*p*
Sex	Female	89	98 (98.9)	1		0.29
	Male	99	86 (96.6)	3.41	0.34–34.19	
Age categories	5–9 years	44	43 (97.7)	1		0.94
	10–15 years	144	141 (97.9)	1.1	0.11–11.01	
Stunting	Non-stunted	128	125 (97.7)	1		0.77
	Stunted	60	59 (98.3)	1.42	0.14–14.19	
Wasted	Non-wasted	179	175 (97.8)	1		
	Wasted	9	9 (100.0)			
Infection Intensity	Light	173	170 (98.3)	1		0.24
	Moderate	15	14 (93.3)	0.25	0.024–2.59	
Co-infections (with STH)	Schistosomiasis only	20	19 (95.0)	1		0.37
	Any co-infection with STH	168	165 (98.2)	2.89	0.28–29.86	

cOR = Crude odd ratio; STH = soil transmitted helminths.

## Data Availability

Data are contained within the article.
